# Supplementary material for: Splice-Junction-Based Mapping of Alternative Isoforms in the Human Proteome
Source: Cell Rep. Author manuscript; Available in PMC 2020 Jan 15. (PMC6961840; doi:10.1016/j.celrep.2019.11.026)
Supplement: 2 [file NIHMS1546469-supplement-2.zip › DF1/PXD010154/Liver/Liver_19_MIB2_HQLLPLVADGAGGDPGPLQLLSRGR.pdf]

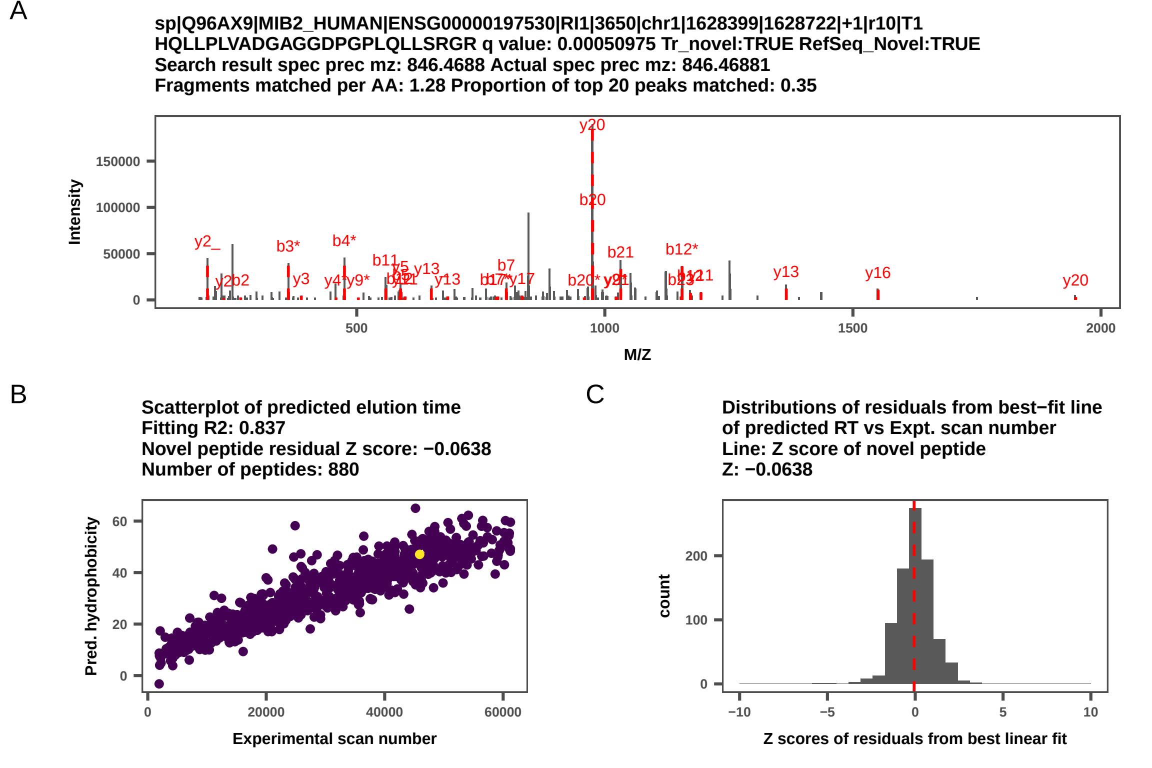
A
sp|Q96AX9|MIB2_HUMAN|ENSG00000197530|RI1|3650|chr1|1628399|1628722|+1|r10|T1
HQLLPLVADGAGGDPGPLQLLSRGR q value: 0.00050975 Tr_novel:TRUE RefSeq_Novel:TRUE
Search result spec prec mz: 846.4688 Actual spec prec mz: 846.46881
Fragments matched per AA: 1.28 Proportion of top 20 peaks matched: 0.35
Intensity
150000
100000
50000
0
y20
b20
y2_
b3*
b4*
b11
b7
b21
b12*
y3
y13
y16
y20
500
1000
1500
2000
M/Z
B
Scatterplot of predicted elution time
Fitting R2: 0.837
Novel peptide residual Z score: −0.0638
Number of peptides: 880
Pred. hydrophobicity
60
40
20
0
0
20000
40000
60000
Experimental scan number
C
Distributions of residuals from best−fit line
of predicted RT vs Expt. scan number
Line: Z score of novel peptide
Z: −0.0638
count
200
100
0
−10
−5
0
5
10
Z scores of residuals from best linear fit
